# Predicting Neonatal Morbidity and Correlations with Maternal and Neonatal Biomarkers in Connection with Fetal Inflammatory Response Syndrome in Premature Births

**DOI:** 10.3390/jcm14186440

**Published:** 2025-09-12

**Authors:** Diana Iulia Vasilescu, Adriana Mihaela Dan, Ion Dragomir, Sorin Liviu Vasilescu, Adrian Vasile Dumitru, Vlad Dima, Monica Mihaela Cîrstoiu

**Affiliations:** 1 Doctoral School, “Carol Davila” University of Medicine and Pharmacy, 020956 Bucharest, Romania; 2 Department of Neonatology, Emergency University Hospital Bucharest, 050098 Bucharest, Romania; 3 Faculty of Medicine, “Carol Davila” University of Medicine and Pharmacy Bucharest, 020956 Bucharest, Romania; 4 Department of Obstetrics and Gynaecology, Emergency University Hospital Bucharest, 050098 Bucharest, Romania; 5 Department of Pathology, Emergency University Hospital Bucharest, 050098 Bucharest, Romania; 6 Filantropia Clinical Hospital, 011177 Bucharest, Romania

**Keywords:** Fetal Inflammatory Response Syndrome, preterm neonates, Interleukin-6, chorioamnionitis, funisitis, placental inflammation, sepsis, neonatal morbidity

## Abstract

**Introduction:** Fetal Inflammatory Response Syndrome (FIRS) is widely acknowledged for its contribution to neonatal morbidity in premature infants. Being a systemic inflammatory process triggered by intrauterine infections or other stimuli, FIRS has gained significant attention due to its complex implications for neonatal adverse outcomes: preterm birth, early onset neonatal sepsis, death or long-term neurodevelopmental impairments. Fetal plasma Interleukin-6 (IL-6) levels above 11 pg/mL define FIRS and serve as an essential biomarker, providing insights into the complex mechanisms underlying this response. This study aims to evaluate the clinical, laboratory, and therapeutic differences between preterm neonates with and without FIRS. **Methods:** A prospective cohort study was conducted, involving 125 preterm neonates with gestational ages between 23 and 37 weeks, who were admitted to the Neonatal Intensive Care Unit (NICU) at the Emergency University Hospital Bucharest between April 2023 and April 2025. Infants were stratified into FIRS and non-FIRS groups based on the measurement of cord blood IL-6 levels greater than 11 pg/mL. Demographic, biochemical, and therapeutic parameters were compared across the two groups. **Results:** Preterm neonates with FIRS had significantly lower birth weight, length, and head circumference, and lower Apgar scores at 1 and 5 min (*p* = 0.001). FIRS was associated with a higher incidence of vaginal delivery, meconium-stained amniotic fluid, and neonatal metabolic imbalances, requiring more respiratory support, longer antibiotic treatment periods, and more blood transfusions (*p* < 0.05). Neonatal complications such as early-onset sepsis (EOS) and late-onset sepsis (LOS), respiratory distress, necrotizing enterocolitis (NEC), intraventricular hemorrhage (IVH), and retinopathy of prematurity (ROP) were significantly more frequent in the FIRS group (*p* ≤ 0.01). Among maternal cervical screening, *Chlamydia trachomatis* was the only pathogen significantly associated with FIRS. **Conclusions:** FIRS in preterm neonates is linked to important perinatal inflammation, adverse short and long-term outcomes, and extensive medical intervention. These findings highlight the value of early identification of intrauterine inflammation and targeted neonatal monitoring strategies. Further studies are needed to explore long-term outcomes and improve diagnostic and therapeutic protocols.

## 1. Introduction

Fetal Inflammatory Response Syndrome (FIRS) represents an intricate, pathophysiologic condition that describes a complex process of activation of the fetal innate immune system [1]. FIRS remains an essential area of investigation within perinatal medicine, as it represents the complex interaction between maternal and fetal immune responses during pregnancy. As a systemic inflammatory process triggered by intrauterine infections or other stimuli, FIRS has gained significant attention due to its serious implications for neonatal adverse outcomes, such as preterm birth, early onset neonatal sepsis, death, and long-term neurodevelopmental impairments [2].

The syndrome is initiated by the activation of the fetal immune system in response to various triggers, such as microbial invasion of the amniotic cavity, maternal inflammation, or placental dysfunction, with the fetal inflammatory response representing a key predictor of neonatal health, affecting both short- and long-term prognosis [3,4]. It has been demonstrated that prenatal inflammation directly alters placental function, subsequently affecting newborn neurodevelopment, particularly motor and cognitive functions [5].

FIRS is characterized by rapid pro-inflammatory cytokine release into the fetal plasma, particularly interleukin 1 (IL-1), interleukin-6 (IL-6), and tumor necrosis factor-alpha (TNFα) associated with a cellular response with increased CD4^+^ cells and neutrophils [1]. IL-6 is a pro-inflammatory cytokine detected in the bloodstream during the early phases of bacterial infection [6]. It is released by a variety of cells (B and T lymphocytes, monocytes) two hours after the onset of bacteriemia, before clinical signs become visible, and it is characterized by a short plasma half-life, approximately 4 to 6 h [7]. When antenatal risk factors are present, such as chorioamnionitis, IL-6 levels can be measured in umbilical cord blood as its level increases significantly, highlighting its important role in the acute immune response [8]. Elevated cord blood IL-6 level often correlates with the tissue changes evidenced in chorioamnionitis and funisitis, connecting the laboratory findings with inflammatory processes at the microscopic level.

Fetal plasma IL-6 above 11 pg/mL defines FIRS and serves as a significant biomarker, unraveling the intricate biological pathways underlying this response [9]. The histologic consequences of FIRS are chorionic vasculitis and funisitis [10,11], while chorioamnionitis is part of the maternal inflammatory response [12]. Funisitis is demonstrated in the presence of a polymorphonuclear leukocyte infiltration in the umbilical cord as a response to intra-uterine infection [2,13]. Term newborns with funisitis have a 12 times higher risk of developing neonatal encephalopathy [14], while cord blood IL-6 > 500 pg/mL leads to a 30-fold increased risk for adverse neonatal outcome [15,16].

In the presence of FIRS, multiple organs can be impaired with the occurrence of subsequent complications: cardiac arrhythmias, patent ductus arteriosus (PDA), gray and white matter injury, intraventricular hemorrhage (IVH), periventricular leukomalacia (PVL), bronchopulmonary dysplasia (BPD), fetal dermatitis, necrotising enterocolitis (NEC) [17,18,19]. It is worth noting that FIRS is not just an isolated physiological process; rather, it reflects a particular dynamic involving maternal–fetal interactions, placental pathologies, and environmental factors. Understanding the prognostic impact of FIRS in preterm neonates is essential for early risk identification and targeted neonatal care.

This research aims to explore the neonatal consequences of FIRS, emphasizing the importance of early diagnosis and innovative therapeutic approaches. By bridging the gap between research and clinical practice, we aspire to provide insight into strategies for decreasing the impact of this condition on both immediate and long-term neonatal outcomes.

The specific objectives are as follows:

- Determining the differences between the FIRS and non-FIRS groups for both neonates and the mothers.
- Assessing the characteristics of the placental and umbilical cord tissue from a histologic approach.
- Identifying the prognosis of neonatal conditions based on FIRS and the cord blood IL-6.

## 2. Materials and Methods

### 2.1. Study Design and Participants

This research was a prospective observational single-center study conducted in the Neonatology Department of Emergency University Hospital Bucharest between April 2023 and April 2025. The study was conducted in accordance with the Ethical Standards of the institutional Research Committee and with the Helsinki Declaration. The research protocol was approved by the Ethics Committee of the hospital (approval no. 13478/21 March 2023). Informed consent was obtained from the parents or legal guardians of all newborns included in the study after providing detailed information regarding the objectives of our research, the medical procedures, and the risks involved.

The study included 125 preterm neonates with a gestational age of less than 37 weeks, delivered in the hospital. This prospective study included neonates born to mothers with clinical suspicion of intrauterine infection or inflammation. The focus on a high-risk maternal population explains the higher prevalence of FIRS observed in our cohort.

The preterm infants were divided into two groups based on the presence or absence of FIRS:

▪ **FIRS group**: 75 preterm neonates who met the diagnostic criteria for FIRS;
▪ **Non-FIRS group**: 50 preterm neonates with no criteria for FIRS.

The inclusion criteria were:

- Preterm birth (<37 weeks of gestation);
- Umbilical cord blood sampling at birth;
- Signed informed consent from the parents or legal guardians.
- Presence of one or more documented maternal infectious risk factors, such as: clinical chorioamnionitis, premature rupture of membranes (PROM) > 18 h, maternal fever >38 °C during labor, positive maternal group B Streptococcus (GBS) colonization, suspected or confirmed intrauterine infection.

Exclusion criteria were:

- Major congenital malformations;
- Incomplete clinical and laboratory data;
- Lack of parental informed consent.

### 2.2. Data Collection

FIRS was diagnosed based on elevated levels of IL-6 in umbilical cord blood, with a cutoff value of > 11 pg/mL, in association with at least one of the following: clinical or histological chorioamnionitis or clinical risk factors for intrauterine inflammation (premature rupture of membranes, maternal fever, or confirmed infection).

Umbilical cord blood samples were collected immediately after delivery under sterile conditions. IL-6 levels were measured using a standardized enzyme-linked immunosorbent assay (ELISA). At birth, in the delivery room, umbilical cord blood gas analysis was also performed for the assessment of fetal acid-base status. Additionally, peripheral blood samples were obtained within the first 24 h of life, including: complete blood count (CBC), complete biochemical profile, inflammatory biomarkers, C-reactive protein (CRP) and procalcitonin (PCT), central cultures, including blood culture and, only when clinically indicated, cerebrospinal fluid (CSF) and peripheral cultures. All clinical data were extracted from medical records.

Placental and umbilical cord samples were collected immediately after birth and sent to the Pathology Department. After fixation in 10% buffered formalin for 24–48 h, the specimens were embedded in paraffin and sectioned at 4 µm thickness. Routine hematoxylin-eosin (H&E) staining was performed for histological evaluation. Selected cases were also processed using immunohistochemical techniques to highlight inflammatory changes and tissue structure. The analysis was completed by pathologists, and representative images were selected for documentation.

### 2.3. Statistical Analysis

Data were collected, processed and analyzed using SPSS, version 25. The data were measured on nominal (categorical) and on continuous scales. The continuous variables were described using means and standard deviations, while the nominal variables were presented as frequencies and percentages. After applying the Shapiro–Wilk Normality test, the differences between the continuous variables depending on the FIRS and non-FIRS assignment were assessed by *t*-tests (*p* value > 0.05) or by Mann–Whitney U tests (emphasized by *). The differences in the distribution of nominal variables were determined by the Chi-square tests. The logistic regression was used to ascertain the likelihood of neonates with FIRS developing specific diseases. More exactly, the logistic regression was employed to determine if FIRS may be a predictor of specific neonatal outcomes. The ROC curve was used to identify the diseases of neonates by taking into consideration the cord blood IL-6 values. The threshold of statistical significance was *p* < 0.05.

## 3. Results

The comparison of neonatal demographic characteristics between the FIRS and non-FIRS groups showed several statistically significant differences, as illustrated in Table 1. Neonates diagnosed with FIRS had significantly earlier gestational age, with a mean of 31.2 weeks of gestation compared to 35.3 weeks of gestation in the non-FIRS group (*p* = 0.001). The study also revealed statistically significant lower birth weight, shorter length, and smaller head circumference compared to those in the non-FIRS group (*p* = 0.001). No statistical differences were found regarding gender. Moreover, Apgar scores at both 1 and 5 min were significantly lower in the FIRS group, indicating a more difficult neonatal transition at birth (*p* = 0.001). A significantly higher proportion of infants in the FIRS group were delivered vaginally (*p* = 0.007), and meconium-stained amniotic fluid was more frequently observed in this group compared to the non-FIRS preterm neonates (*p* = 0.008), potentially reflecting intrauterine fetal distress.

**Figure 1:**
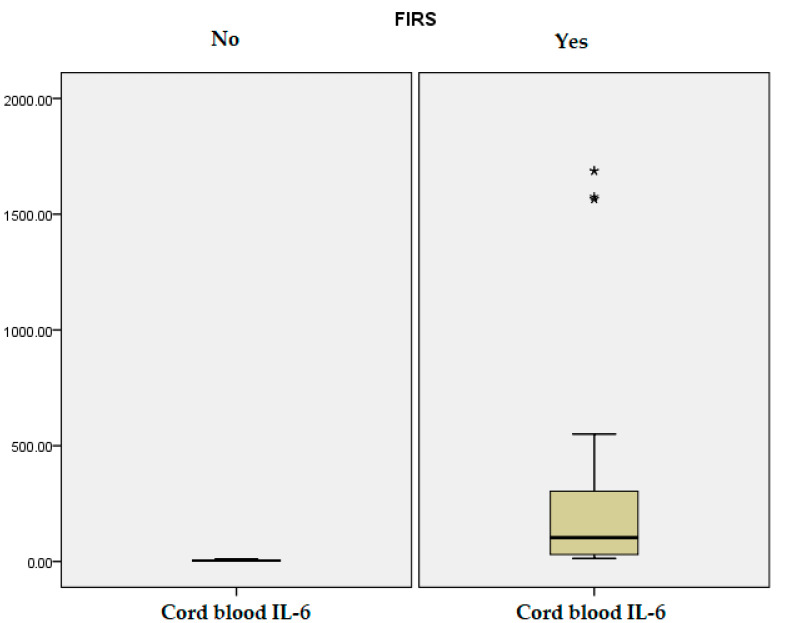
Cord blood IL-6 distribution. “*” represents values outside the typical range.

**Figure 2:**
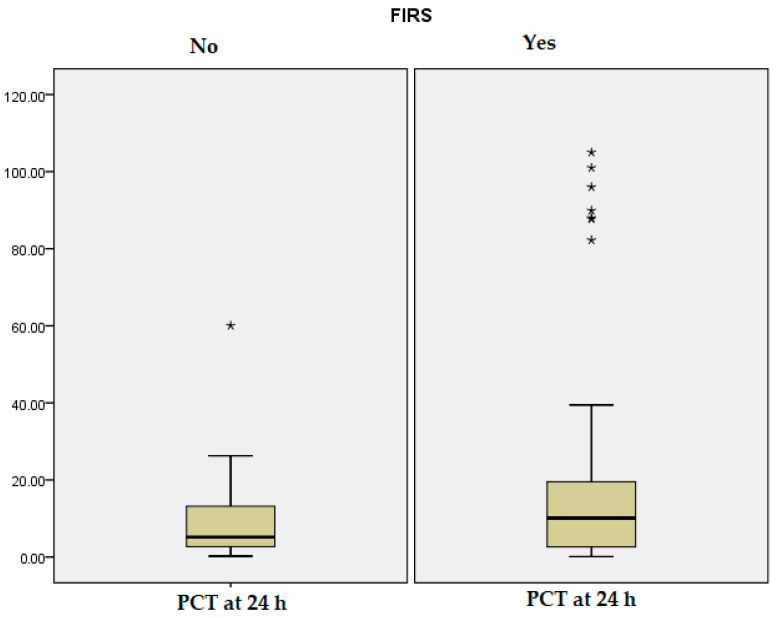
The distribution of PCT at 24 h. “*” represents values outside the typical range.

Neonatal laboratory parameters within the first 24 h of life showed several significant differences between the FIRS and non-FIRS groups, as illustrated in Table 1. Newborns in the FIRS group had lower arterial blood pH (*p* = 0.04) and base excess values (*p* = 0.001), suggesting a higher incidence of metabolic acidosis. Cord blood IL-6 and PCT levels were significantly elevated in the FIRS group compared to non-FIRS neonates (*p* = 0.001, *p* = 0.008), highlighting their potential role as early markers of systemic inflammation, as it can be observed in Figure 1 and Figure 2. Blood glucose levels were also significantly higher among FIRS infants (*p* = 0.004). No statistically significant differences were observed between the groups in terms of CRP levels, leukocyte count, neutrophils, or platelet count (*p* > 0.05 for all), suggesting that these standard hematologic markers may have limited early diagnostic value for FIRS in preterm neonates.

Therapeutic approaches varied considerably between neonates with and without FIRS, reflecting differences in both disease severity and clinical evolution, as shown in Table 2.

Mechanical ventilation was required significantly more often in the FIRS group, both invasive (*p* = 0.008) and non-invasive (*p* = 0.001) forms, indicating a greater degree of respiratory compromise at birth. Neonates in the FIRS group also had longer durations of antibiotic therapy (*p* = 0.001). Both their length of stay in the neonatal intensive care unit (NICU) (*p* = 0.001) and overall hospitalization (*p* = 0.001) were significantly longer compared to the non-FIRS group. Additional interventions such as phototherapy, surfactant, inotrope and caffeine administration (*p* = 0.001) were more frequently required in the FIRS group. Furthermore, red blood cell transfusions and fresh frozen plasma (FFP) administration were significantly more common among FIRS neonates (*p* = 0.001), possibly reflecting the degree of systemic inflammation and coagulopathy often associated with the syndrome.

Analysis of maternal laboratory and clinical parameters at delivery revealed several statistically significant differences between the FIRS and non-FIRS groups, as can be observed in Table 3. PROM was significantly more frequent among mothers of neonates with FIRS (*p* = 0.01), suggesting a higher risk of invasive intrauterine infection. Maternal leukocyte count, ANC, and neutrophil percentage were all significantly elevated in the FIRS group (*p* < 0.03 for all), while the percentage of lymphocytes was lower (*p* = 0.005). These findings are consistent with the existence of a maternal inflammatory response. Additionally, maternal CRP levels were significantly higher in the FIRS group (*p* = 0.01).

Regarding prenatal management, a significantly higher proportion of mothers in the FIRS group received intrapartum antibiotic therapy (*p* = 0.02) or antenatal corticosteroids (*p* = 0.01), possibly reflecting the clinicians’ response to signs of inflammation or preterm labor risk.

**Figure 3:**
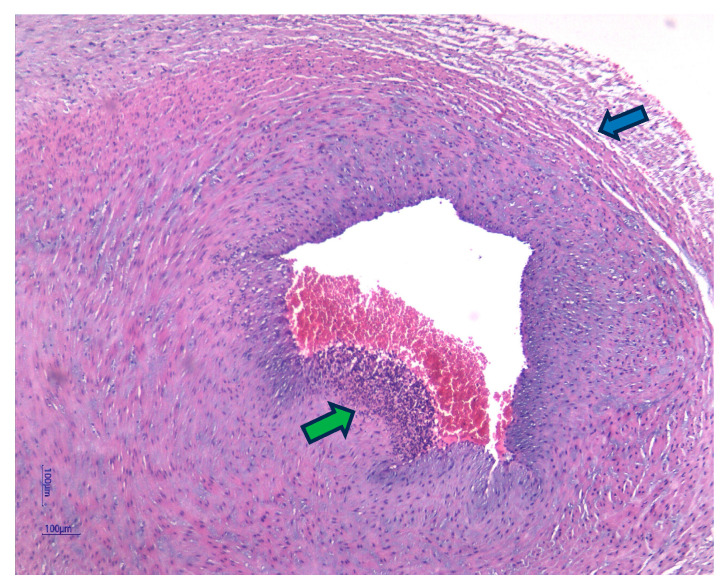
Funisitis inflammation (blue arrow) within Wharton’s Jelly and acute arteritis (green arrow). Hematoxylin and eosin (HE) stain, objective 10×. (image obtained using placental samples from our patient cohort and processed within the institutional pathology laboratory).

**Figure 4:**
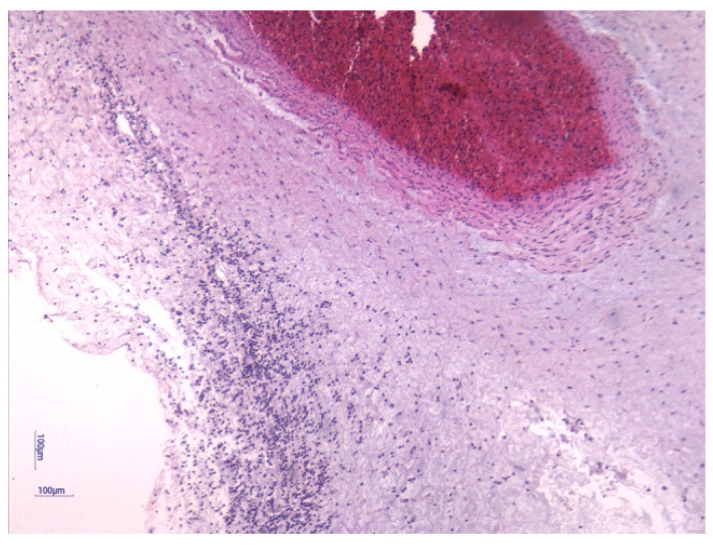
Peripheral funisitis inflammation is located at the radial periphery or external surface of the umbilical cord. The HE stain 10× objective. (image obtained using placental samples from our patient cohort and processed within the institutional pathology laboratory).

**Figure 5:**
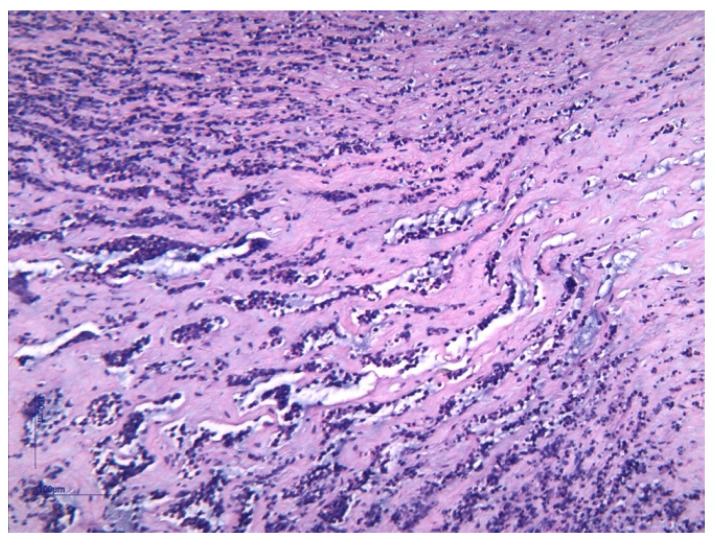
Microscopic detail of the acute inflammatory infiltrate within Wharton’s jelly. The HE stain 20× objective. (image obtained using placental samples from our patient cohort and processed within the institutional pathology laboratory).

**Figure 6:**
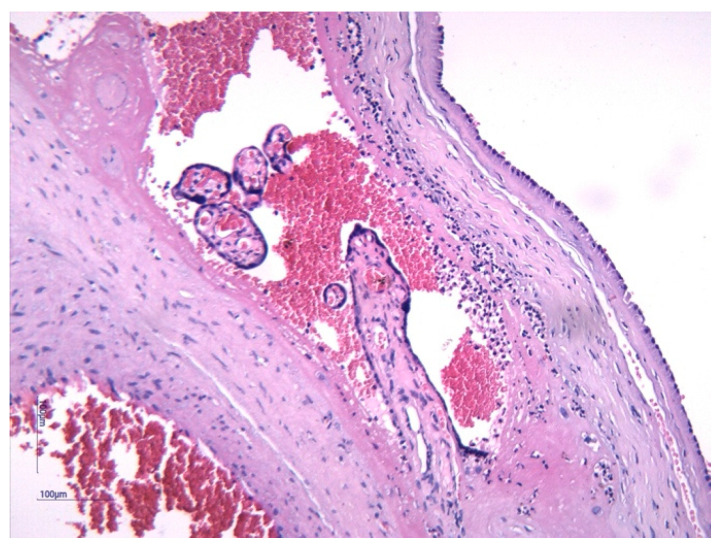
Stage 1, grade 1 Chorioamnionitis. The HE stain 20× objective. (image obtained using placental samples from our own patient cohort and processed within the institutional pathology laboratory).

**Figure 7:**
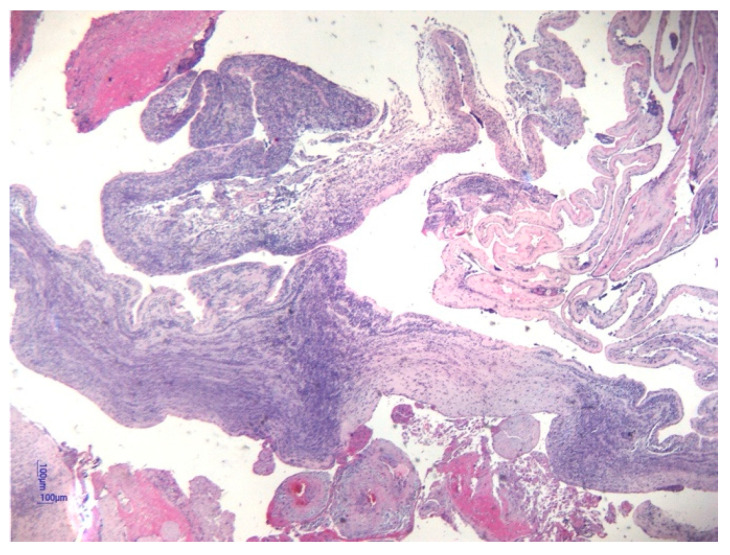
Stage 3, grade 2 Corioamnionitis following the Redline and Amsterdam criteria. HE uses a 4× objective. (image obtained using placental samples from our patient cohort and processed within the institutional pathology laboratory).

**Figure 8:**
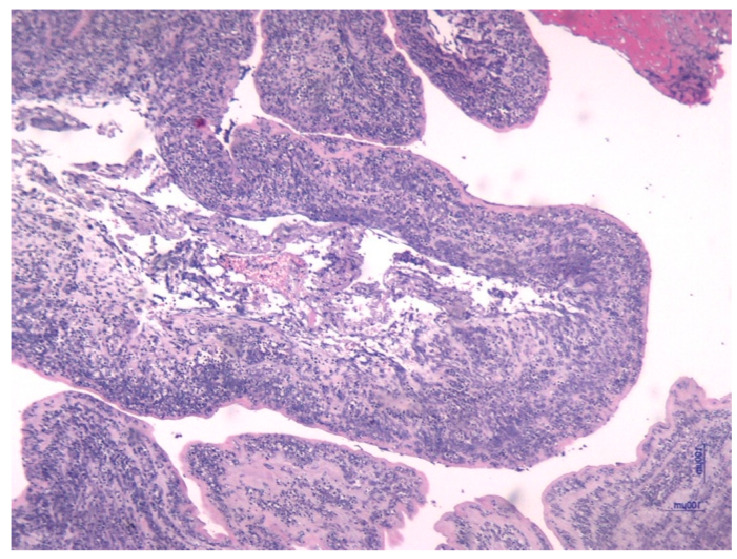
Necrotising chorioamnionitis. Membrane necrosis and sloughing of amniotic epithelium are seen in advanced cases. HE stain a 10× objective. (image obtained using placental samples from our patient cohort and processed within the institutional pathology laboratory).

Histological analysis of placental and umbilical cord tissues showed a significantly higher incidence of chorioamnionitis (*p* = 0.001) and funisitis (*p* = 0.001) in the FIRS group, confirming the intrauterine inflammatory nature of the syndrome. The following images illustrate the histopathological features of funisitis (Figure 3, Figure 4 and Figure 5) and chorioamnionitis (Figure 6, Figure 7 and Figure 8), inflammatory changes that were strongly associated with FIRS in this study.

Maternal cervical screening during pregnancy revealed a significantly higher prevalence of *Chlamydia trachomatis* infection in the FIRS group compared to the non-FIRS group (*p* = 0.02), whereas no statistically significant differences were found for Group B Streptococcus (GBS), *Ureaplasma* spp., *Klebsiella pneumoniae*, or *Escherichia coli* (*p* > 0.05 for all), as shown in Table 4.

Findings underscore the extensive and multi-organ impact of the Fetal Inflammatory Response Syndrome in preterm infants, with significant implications for neonatal care and prognosis.

Neonates with FIRS were significantly more likely to develop a range of short and long-term complications during hospitalization compared to their non-FIRS counterparts, as seen in Table 5. The incidence of EOS and LOS was higher in the FIRS group (*p* < 0.002 for both), emphasizing the increased infectious vulnerability associated with intrauterine inflammation. Respiratory complications were also significantly more frequent, including respiratory distress (*p* = 0.001) and BPD (*p* = 0.001), suggesting both acute and chronic pulmonary impact of fetal systemic inflammation. Gastrointestinal complications were more common in the FIRS group as well. The incidence of NEC (*p* = 0.04) and feeding intolerance (*p* = 0.001) was significantly higher, indicating altered gut immunity and immaturity. Cardiac involvement was reflected by a significantly higher rate of patent ductus arteriosus (PDA) requiring intervention (*p* = 0.001). Furthermore, central nervous system complications were more prevalent, with higher rates of IVH (*p* = 0.001) and seizures (*p* = 0.003), potentially correlating with both inflammatory injury and prematurity. Other significant findings in the FIRS group included a higher incidence of ROP (*p* = 0.001) and hemorrhagic events (*p* = 0.001), reflecting greater clinical instability.

These findings underscore the extensive and multi-organ impact of the fetal inflammatory response syndrome in preterm infants, with significant implications for neonatal care and prognosis.

Table 6 presents the likelihood prevalence (OR) of specific neonatal conditions, with FIRS being the predictor. Newborns diagnosed with FIRS show significantly increased risks of adverse outcomes such as EOS, respiratory distress, BPD, IVH, and PDA compared to those without FIRS.

Table 7 summarizes the IL-6 cut-off values and corresponding AUCs for predicting specific neonatal conditions. These findings show the diagnostic performance of IL-6 in identifying at-risk premature infants and confirm its potential role in early diagnosis.

To explore the influence of gestational age on neonatal outcomes, we performed a second analysis and stratified the study population into three gestational age categories: 22–27 weeks, 28–31 weeks, and 32–36 weeks, as shown in Table 8.

Due to the absence of non-FIRS neonates in the most premature group (22–27 weeks) and the limited number of controls in the 28–31 weeks group, statistical comparisons in these categories were limited. However, descriptive data indicated that neonates with FIRS consistently presented with lower Apgar scores, higher inflammatory markers (including IL-6 and CRP), and increased rates of respiratory and neurological complications. In the 32–36 weeks subgroup—where case distribution allowed more meaningful comparisons—neonates with FIRS had significantly lower birth weight, length, and head circumference, as well as lower Apgar scores at 1 and 5 min (*p* < 0.01 for all). They also showed significantly elevated levels of IL-6 in umbilical cord blood (*p* = 0.001), and higher rates of early-onset sepsis (*p* = 0.001), respiratory distress (*p* = 0.005), feeding intolerance (*p* = 0.002), seizures (*p* = 0.04), and bleeding (*p* = 0.04), compared to the non-FIRS group. Notably, the presence of funisitis and chorioamnionitis was significantly higher in the FIRS group compared to the non-FIRS group (*p* = 0.005 and *p* = 0.006, respectively). These findings reinforce the association between FIRS and adverse neonatal outcomes, particularly in late preterm infants.

## 4. Discussion

During pregnancy, the inflammatory status is strictly regulated, given that an effective immune response is crucial for the protection of both the mother and the fetus from pathogens, but excessive inflammation can be harmful to the developing fetus [20]. The consequence of immune system activation is the release of pro-inflammatory cytokines, such as interleukin-1β (IL-1β), IL-6, and TNF-α. As a result, direct damage to fetal organ development can occur, potentially resulting in long-term neurodevelopmental disabilities [21].

The placenta, a critical interface between the mother and the fetus, plays a vital role in modulating maternal and fetal immune responses [22]. Moreover, placental inflammation can disrupt its function, leading to impaired nutrient and oxygen transport to the fetus, further exacerbating fetal injury. Furthermore, fetal inflammatory response can trigger a cascade of events that ultimately lead to preterm birth [23].

Our study aimed to investigate the neonatal impact of FIRS among preterm infants, focusing on clinical, laboratory, and outcome parameters, and to correlate these findings with maternal and placental inflammatory markers. Results demonstrate that preterm neonates with FIRS exhibit a distinct clinical profile, marked by lower birth weight, reduced Apgar scores, and a significantly higher incidence of respiratory, infectious, gastrointestinal, neurological, and hematologic complications. These infants required more frequent and prolonged medical interventions, including invasive ventilation, antibiotics, blood products, and corticosteroids. Notably, elevated PCT levels and metabolic acidosis within the first 24 h of life support the presence of early systemic inflammation and physiological compromise in the FIRS cohort. Considering these observations, a comparison with previously published data offers further context and insight.

In this study, regarding neonatal demographics, the FIRS group showed significantly lower gestational age, reduced birth weight, lower Apgar scores at both 1 and 5 min, and a higher incidence of meconium-stained amniotic fluid, confirming the statistical relevance of these parameters. This strong concordance with published data supports a shared pathogenic pathway: fetal inflammation likely contributes to perinatal stress, manifesting as unfavorable birth demographic indicators. Multiple studies in preterm infants with confirmed FIRS have consistently reported associations between fetal inflammation and adverse birth demographic indicators. After analyzing an extremely preterm cohort, Salas et al. reported a significantly lower median gestational age in the FIRS group (25 weeks vs. 26 weeks in non-FIRS, *p* = 0.002) along with a higher prevalence of severe IVH, though birth weight was similar. This emphasized the compounded vulnerability of earlier births [24]. A retrospective cohort of 176 preterm infants (24–36 weeks of gestation) analyzed by Hofer et al. demonstrated that neonates with FIRS (cord IL-6 > 11 pg/mL) had significantly lower gestational age (median 30.7 vs. 33.0 weeks; *p* < 0.001) and reduced birth weight (1419 vs. 1878 g; *p* < 0.001) compared to non-FIRS infants. Additionally, severe IVH and EOS were significantly more frequent in the FIRS group [16]. Recent evidence, including multivariate cohort analyses and meta-analyses, confirms that FIRS is an independent predictor of severe neonatal morbidity and mortality (e.g., EOS, BPD, IVH, PVL) in preterm infants, even after adjusting for gestational age and other confounders [2].

Concerning the neonatal laboratory results, unlike our findings, a study conducted by Romero et al. showed no differences regarding pH values in preterm infants with FIRS compared with the non-FIRS group [25]. Regarding the hematopoietic response in FIRS, Dulay et al. demonstrated in their study on inflammation and preterm birth that elevated levels of nucleated red blood cells (NRBCs) were significantly associated with intrauterine inflammation, suggesting a compensatory erythropoietic response to fetal hypoxia and systemic insult [26]. Circulating NRBC count is now recognized as a robust indicator of fetal inflammatory response in preterm infants, correlating with intra-amniotic inflammation and early-onset neonatal sepsis (EONS), even after adjusting for gestational age and absence of hypoxia [27,28]. Consistent with these findings, our study also identified a statistically significant difference in hematocrit values within the FIRS group, further supporting the hypothesis of altered hematopoiesis in the context of fetal inflammatory activation.

The extensive multi-organ impact of FIRS and increased resource utilization in NICU settings support the need for early identification and targeted management. The use of cord blood IL-6 and PCT (rapid rise within 4–8 h postpartum with 92% sensitivity and 97% specificity) may demonstrate their relevance to early diagnosis and guiding antibiotic stewardship [29]. Our study further confirms these associations and highlights PCT’s emerging role as a more sensitive early biomarker than traditional markers such as CRP, consistent with previous publications on neonatal sepsis biomarkers [29,30].

In terms of maternal laboratory findings, a prospective study conducted by LeRay et al. on 121 women with PROM between 24 and 34 + 0 weeks of gestation evaluated the relationship between maternal inflammatory markers, specifically white blood cell count (WBC), CRP, IL-6, and the presence of histological chorioamnionitis (HCA). The authors reported that 44.7% of cases had histologically confirmed HCA, and significantly elevated levels of WBC, CRP, and IL-6 were observed in these patients before delivery (*p* < 0.001) [31]. These findings are in accordance with our results, where maternal inflammatory biomarkers such as CRP, leukocyte count, and ANC showed statistically significant differences in the FIRS group. The elevated maternal inflammatory profile in our cohort further supports the role of systemic maternal immune activation in the pathogenesis of fetal inflammation and its expression (chorioamnionitis and funisitis). Notably, the aforementioned prospective study highlighted the limited predictive accuracy of these markers alone. Our findings underscore their value when interpreted in association with neonatal outcomes and placental pathology.

The combination of placental histopathology (chorioamnionitis/funisitis) and maternal inflammatory markers (leukocytosis, CRP) further enhances risk assessment [32]. A study conducted by Yoon BH et al. investigated the association between funisitis, umbilical cord IL-6 levels, intra-amniotic infection, and neonatal sepsis in 315 preterm births. Funisitis was identified in 25% of cases and was significantly associated with elevated cord blood IL-6 levels, lower gestational age, increased rates of microbial invasion of the amniotic cavity, and congenital neonatal sepsis. An IL-6 threshold ≥17.5 pg/mL showed moderate sensitivity and specificity for detecting funisitis. Even after adjusting for gestational age, funisitis remained a strong predictor of neonatal sepsis (OR 7.2) [33]. The findings support funisitis as a histological marker of fetal inflammatory response and adverse neonatal outcomes. According to our findings, at a cord blood IL-6 threshold ≥ 11.92 pg/mL, in the FIRS group, we identified a sensitivity of 88.1% for chorioamnionitis and 89.2% for funisitis and a specificity of 45.8% for chorioamnionitis and 47.7% for funisitis.

Our detection of *Chlamydia trachomatis*, while other organisms were not significant, as a statistically significant infection in the cervical screening of affected mothers, may suggest a potential trigger for intrauterine inflammation. Epidemiologic data increasingly link antenatal *C. trachomatis* infection with adverse pregnancy outcomes such as preterm birth, low birthweight and neonatal morbidity, although results are heterogeneous across settings and study designs [34]. The literature remains limited with respect to direct, pathogen-specific pathways leading to FIRS and many reports highlight the need for further mechanistic and longitudinal studies. Some studies suggest that coinfections (e.g., bacterial vaginosis) and host immune factors may modify the effect of *C. trachomatis* on pregnancy outcomes, which could explain variability between studies [35,36]. Therefore, while our findings are consistent with a possible role of *C. trachomatis* in triggering fetal inflammatory pathways, confirmation in larger, prospective cohorts with molecular and immunologic profiling is warranted. The association between maternal immune activation and neonatal systemic response reinforces the importance of early detection and targeted management of subclinical infections during pregnancy. Notably, even subclinical maternal infections may initiate a fetal inflammatory cascade, highlighting that maternal microbial invasion can be both overt and silent.

Concerning the neonatal adverse outcomes, in concordance with prior meta-analyses conducted by Tang et al., our findings underscore that FIRS significantly increases the risk of severe neonatal morbidities, including EOS, BPD, IVH, PVL, respiratory distress syndrome (RDS), and even mortality [37]. Our study demonstrated that the presence of FIRS was associated with a more complicated respiratory course, characterized by prolonged mechanical ventilation and increased need for surfactant administration. Infants in the FIRS group were of earlier gestational age, a factor likely contributing to the increased need for respiratory support. Multiple studies have reported elevated levels of IL-6 in the cord blood of preterm infants with RDS, particularly among those who later progressed to develop BPD [38]. For instance, one meta-analysis aggregating data from over 1.100 neonates reported the relative risk for BPD as 5.9 and for death as 7.0 compared to non-FIRS infants [39]. Our findings are generally consistent with previous studies, including the work by McAdams et al., which also highlighted the association between FIRS and neonatal respiratory complications. However, our study provides additional insights into early neonatal adaptation (e.g., Apgar scores, delivery mode, meconium-stained fluid), emphasizing the clinical impact of FIRS beyond structural morbidity [40].

In another prospective observational study from Austria. Hofer et al. evaluated 176 preterm neonates and demonstrated that FIRS was significantly associated with adverse neonatal outcomes, including severe morbidity and mortality. Notably, FIRS correlated strongly with IVH, EOS, BPD, and death, particularly among infants < 32 weeks’ gestational age. The median IL-6 concentration in the FIRS group was markedly elevated (51.8 pg/mL). and regression analysis confirmed its predictive value for poor neonatal prognosis [16]. These findings are consistent with our results, where FIRS was significantly associated with a higher incidence of early- and LOS, IVH, BPD, NEC and neonatal death. The parallel between IL-6-driven inflammatory activation and the spectrum of neonatal complications supports the use of inflammatory biomarkers as early prognostic indicators in this high-risk population.

Previous studies by multiple authors have established a link between systemic inflammation and the development of white matter lesions, particularly in the context of preterm birth [41]. A 2025 study in the *Journal of Neuroinflammation* demonstrated that prenatal inflammation, especially when combined with postnatal hyperoxia, significantly exacerbates white matter injury (evidenced by myelination deficits and microglial activation) in a neonatal rodent model, highlighting the synergistic impact of systemic inflammation on immature oligodendrocytes [42]. Another review in *Cellular and Molecular Neurobiology* further identified inflammation as a critical driver of diffuse white matter injury in preterm infants, characterized by arrested oligodendrocyte maturation and gliosis [43]. These findings are in agreement with our outcomes, where FIRS was associated with increased rates of IVH, seizures, and retinopathy—conditions often coexistent with diffuse white matter damage. The concordance across experimental and clinical data emphasizes that systemic fetal inflammation plays a pivotal role in perinatal white matter pathology, which may predispose preterm infants to long-term neurodevelopmental impairment.

In a cohort of 838 preterm infants born before 30 weeks of gestation, Strunk et al. demonstrated that HCA was associated with a significantly high risk of EOS, but with a reduced risk of LOS. The findings suggest that perinatal inflammation may promote immune maturation and confer partial protection against LOS in very preterm neonates [44]. Nevertheless, current evidence on this topic remains limited. Our findings contribute to this area by demonstrating a statistically significant association between FIRS and both EOS and LOS, supporting the role of fetal inflammation as a relevant perinatal risk factor.

This research strengthens the understanding that FIRS represents a distinct clinical condition in preterm neonates, characterized by a significant risk of morbidity and a greater need for intensive medical care. Moreover, the correlation between maternal inflammation, placental histopathology (chorioamnionitis and funisitis), and the presence of FIRS validates the intrauterine origin of this systemic response. These results highlight the importance of prenatal screening, early recognition of risk factors linked to FIRS, along with individualized postnatal monitoring strategies to reduce complications and improve outcomes in this vulnerable population.

## 5. Study Limitations and Future Directions of Research

This study is limited by its small number of patients included and the single-center data collection, which may affect the generalizability of the findings. In addition, we consider that another potential limitation of this study is that the preterm infants were not stratified by gestational age in a multivariate analysis, which could have influenced outcome variability and contributed to potential biases in interpreting the impact of FIRS across different degrees of prematurity. More exactly, our analysis was limited by the inability to perform multivariate adjustments for gestational age, due to uneven subgroup sizes and a lack of matched controls in the earlier gestational age categories. However, this reflects the natural distribution of the population under investigation, rather than a recruitment bias. Such differences are common in clinical and epidemiological research, where group sizes often differ due to real-world prevalence [45]. Consequently, a small group of preterm infants, born near term without risk factors, was intentionally excluded, and an overrepresentation of FIRS cases was identified. As such, the findings may not be generalizable to all preterm infants, but they provide clinically relevant insights for populations at increased risk of fetal inflammation. Further, to partially address this limitation, while statistical testing was not feasible in the more premature groups, the 32–36 weeks subgroup with FIRS demonstrated significantly worse clinical and inflammatory profiles with poorer adaptation and clinical outcomes than their non-FIRS counterparts. These findings suggest that the fetal inflammatory response contributes to neonatal morbidity independently, beyond the effects of prematurity alone.

Additionally, our analysis was deliberately restricted to a population at higher risk for adverse outcomes, including only neonates with documented antenatal maternal risk factors or preterm labor. Future prospective studies with larger cohorts are needed to validate specific biomarkers for FIRS and to evaluate the long-term neurodevelopmental outcomes of affected neonates.

## 6. Conclusions

FIRS shows diverse clinical and biological manifestations, and it is influenced by the timing, intensity, and persistence of intrauterine inflammation, as well as the fetus’s genetic predisposition. Measuring IL-6 in umbilical cord blood at preterm birth helps identify newborns at risk and supports personalized care. Our study confirms that FIRS is linked to significant inflammation, adverse short and long-term outcomes, and increased medical interventions. Early detection and targeted neonatal monitoring are essential for reducing the negative impact of FIRS on both fetal and neonatal health. Continued research is needed to better understand its mechanisms and improve care strategies and therapeutic protocols.

## Figures and Tables

**Table 1:** Demographic and baseline characteristics of the newborns and laboratory parameters in premature infants at birth and within the first 24 h of life.

Variable		N = 125		*p*-Value
		FIRS (n = 75)	Non-FIRS (n = 50)	
Gestational age (weeks) (SD)		31.17 (3.86)	35.32 (1.91)	0.001
Birth weight (grams) (SD)		1660 (1147.50–2292.50) *	2600 (2200–2930) *	0.001
Length at birth (cm) (SD)		40.24 (5.68)	46.37 (3.54)	0.001
Head circumference (cm) (SD)		28.19 (3.91)	32.19 (2.31)	0.001
APGAR 1 min		6.50 (4.00–8.00) *	9.00 (8.00–9.00) *	0.001
APGAR 5 min		8.00 (7.00–9.00) *	9.00 (8.50–9.00) *	0.001
Gender (n = 125)	Male	41 (32.80%)	32 (25.6%)	0.30
	Female	34 (27.20%)	18 (14.40%)	
Vaginal delivery		23 (82.14%)	5 (17.86%)	0.007
Intrauterine growth restriction (n = 16)		13 (81.25%)	3 (18.75%)	0.06
Meconium-stained amniotic fluid (n = 14)		13 (92.85%)	1 (7.15%)	0.008
Umbilical Astrup—pH		7.28 (0.10)	7.32 (0.09)	0.04
Umbilical Astrup—Base excess		−5.19 (4.03)	−2.36 (3.52)	0.001
Umbilical cord IL-6 (pg/mL)		259.65 (43.40)	4.13 (2.30)	0.001
IL-6 at 24 h (pg/mL)		47.84 (6.39)	21.94 (3.41)	0.01
CRP at 24 h (mg/L)		6.00 (15.88)	2.20 (2.65)	0.09
PCT at 24 h (ng/mL)		20.44 (27.46)	9.32 (11.05)	0.008
Leucocyte (/µL)		14,366.93 (10061.90)	14,534 (4710.15)	0.91
Hematocrit level (%)		44.99 (7.74)	48.52 (6.03)	0.008
RDW (%)		16.86 (2.46)	17.51 (1.55)	0.10
Platelet count (/µL)		256,000 (86516.36)	279,340 (70,520.11)	0.11
ANC (/µL)		6.10 (4.17–9.75) *	8.10 (5.40–12.05) *	0.07
Lymphocyte (%)		30.05 (20.80–41.20) *	28.30 (21.50–36.75) *	0.70
Lymphocyte absolute number (/µL)		3.96 (1.96)	4.22 (1.45)	0.43
NLR		2.59 (2.67)	2.38 (1.56)	0.61
ALT (U/L)		9.24 (8.70)	11.69 (9.10)	0.13
AST (U/L)		57.73 (31.86)	49.78 (33.62)	0.18
Blood glucose (mg/dL)		74.91 (44.29)	55.80 (15.94)	0.004
Positive blood culture (n = 4)		3 (75%)	1 (25%)	0.95

Note: Data are expressed as mean (SD), median, and interquartile range for (*) or n (%). Abbreviations: RDW (Red Cell Distribution Width), ANC (Absolute Neutrophil Count), NLR (Neutrophil-to-Lymphocyte Ratio), ALT (Alanine Aminotransferase), AST (aspartate aminotransferase).

**Table 2:** Neonatal interventions during NICU.

Variable	N = 125		*p*-Value
	FIRS (n = 75)	Non-FIRS (n = 50)	
Hours of noninvasive ventilation	107.64 (182.60)	12.68 (35.59)	0.001
Hours of invasive ventilation	99.80 (245.80)	4.80 (30.66)	0.008
Oxygen therapy hours	223.71 (370.51)	31.30 (87.37)	0.001
Parenteral nutrition days	16.76 (14.81)	3.08 (4.80)	0.001
Tube feeding days	20.40 (21.91)	2.50 (6.91)	0.001
Antibiotic therapy days	16.23 (15.16)	2.92 (3.88)	0.001
NICU days	20.25 (18.18)	3.16 (4.16)	0.001
Total hospitalization days	30.00 (12.00–50.00) *	5.00 (3.00–13.00) *	0.001
Phototherapy hours	27.63 (23.76)	10.40 (14.48)	0.001
Caffeine treatment days	185.15 (19.98)	2.74 (6.53)	0.001
Fresh Frozen Plasma transfusion	0.56 (1.03)	0.041 (0.19)	0.001
Red blood cell transfusion	1.21 (2.00)	0.001 (0.001)	0.001
Surfactant administration (n = 28)	27 (96.42%)	1 (3.58%)	0.001
Need for inotrope (n = 31)	30 (96.77%)	1 (3.23%)	0.001
Human Immunoglobulin (n = 28)	27 (96.42%)	1 (3.58%)	0.001
Sedation (n = 32)	30 (93.75%)	2 (6.25%)	0.001
Corticotherapy (n = 21)	19 (90.47%)	2 (9.53%)	0.002

Note: Data are expressed as mean (SD), median and interquartile range for (*) or n (%).

**Table 3:** Maternal characteristics and laboratory findings at delivery.

Variable		N = 125		*p*-Value
		FIRS (n = 75)	Non-FIRS (n = 50)	
Mother’s age		31.24 (6.94)	32.34 (5.28)	0.34
PROM hours		75.51 (195.70)	2.76 (8.20)	0.01
Leucocyte count (/µL)		13,440.13 (5932.24)	11,074 (3520.13)	0.01
Neutrophil %		78.10 (72.80–84.10) *	73.85 (69.67–79.17) *	0.006
ANC level (/µL)		10.91 (7.19)	8.48 (3.81)	0.03
Lymphocyte %		14.61 (5.83)	17.86 (6.90)	0.005
Lymphocyte count (/µL)		1.43 (0.68)	1.98 (1.08)	0.04
Platelet count (/µL)		249,822.66 (78,263.76)	228,360 (76,693.33)	0.13
CRP (mg/L)		25.47 (38.81)	11.20 (21.27)	0.01
Fibrinogen (mg/dL)		471 (423–629) *	476 (446.25–588.25) *	0.59
ALT (U/L)		15 (11–21) *	12 (8–17.25) *	0.18
AST (U/L)		20 (17–31) *	18 (15–25) *	0.09
Antibiotic therapy during pregnancy (n = 89)		48 (53.93%)	41 (46.07%)	0.02
Antenatal corticosteroids (n = 41)		31 (75.60%)	10 (24.40%)	0.01
Corioamnionitis (n = 42)		37 (88.1%)	5 (11.9%)	0.001
Funisitis (n = 37)		33 (89.2%)	4 (10.8%)	0.001
Smoking (n = 48)		34 (70.84%)	14 (29.16%)	0.051
Socio-economic status (n = 125)	low	30 (24%)	10 (8%)	0.059
	middle	23 (18.4%)	22 (17.59%)	
	high	22 (17.59%)	18 (14.42%)	
Residential area (n = 125)	Urban	33 (26.40%)	32 (25.6%)	0.08
	Rural	42 (33.6%)	18 (14.40%)	
Marital status: married (n = 85)		48 (56.47%)	37 (43.53%)	0.24

Note: Data are expressed as mean (SD), median and interquartile range for (*) or n (%).

**Table 4:** Maternal cervical screening during pregnancy.

Variable	FIRS (n = 75)	Non-FIRS (n = 50)	*p*-Value
Group B Streptococcus (n = 36)	11 (30.55%)	25 (69.45%)	0.45
*Ureaplasma* spp. (n = 16)	9 (56.25%)	7 (43.75%)	0.92
*Chlamydia trachomatis* (n = 10)	9 (90%)	1 (10%)	0.02
*Klebsiella pneumoniae* (n = 8)	6 (75%)	2 (25%)	0.29
*Escherichia coli* (n = 31)	19 (61.29%)	12 (38.71%)	0.54

**Table 5:** Neonatal pathology.

Variable	FIRS (n = 75)	Non-FIRS (n = 50)	*p*-Value
EOS (n = 65)	62 (95.38%)	3 (4.62%)	0.001
LOS (n = 13)	13 (100%)	0 (0%)	0.002
Respiratory distress (n = 66)	55 (83.33%)	11 (16.67%)	0.001
BPD (n = 19)	18 (94.73%)	1 (5.27%)	0.001
NEC (n = 6)	6 (100%)	0 (0%)	0.04
PDA (n = 39)	33 (84.62%)	6 (15.38%)	0.001
ROP (n = 15)	15 (100%)	0 (0%)	0.001
IVH/PVL (n = 39)	33 (84.62%)	6 (15.38%)	0.001
Apnea (n = 53)	45 (84.90%)	8 (15.10%)	0.001
Feeding intolerance (n = 67)	52 (77.61%)	15 (22.39%)	0.001
Seizures (n = 12)	12 (100%)	0 (0%)	0.003
Bleeding (n = 15)	15 (100%)	0 (0%)	0.001
Death (n = 4)	4 (100%)	0 (0%)	0.09

**Table 6:** Logistic regression analysis of FIRS for the specific neonatal outcomes.

Variable	OR (95% CI)	*p*-Value
EOS	74.71 (20.13–277.29)	0.001
Respiratory distress	9.75 (4.19–22.63)	0.001
BDP	15.47 (1.99–120.14)	0.009
PDA	5.76 (2.19–15.15)	0.001
IVH	5.76 (2.19–15.15)	0.001

Note: OR—odds ratio, CI—confidence interval.

**Table 7:** Analysis of the cord blood IL-6 ROC curves for the prediction of the neonatal outcomes.

Variable	AUC	95% CI	*p*-Value	Cut-Off	Sensitivity %	Specificity %
EOS	0.93	0.89–0.98	0.001	11.92	95.4%	21.7%
LOS	0.85	0.75–0.94	0.001	9.92	100%	56.3%
BPD	0.78	0.67–0.89	0.001	13.38	94.7%	52.8%
NEC	0.77	0.60–0.53	0.02	13.53	100%	56.3%
PDA	0.70	0.60–0.79	0.001	13.76	84.6%	45.3%
ROP	0.80	0.69–0.90	0.001	14.22	100%	50.9%
IVH	0.74	0.65–0.83	0.001	14.62	84.6%	43.0%
Death	0.85	0.64–1.00	0.01	17.32	75%	50.4%

Note: ROC: receiver operating characteristic, CI: confidence interval; AUC: Area under the curve.

**Table 8:** Neonatal outcomes and inflammatory markers stratified by gestational age and FIRS status. Data are presented as mean (standard deviation) for continuous variables and n (%) for categorical variables. Statistical comparisons were performed where feasible. In the 22–27 weeks group, no non-FIRS cases were available. Comparisons in the 28–31 weeks group should be interpreted with caution due to the very small size of the non-FIRS subgroup (n = 2). Statistically significant *p*-values (<0.05) are shown in bold.

Variable	GA = 22–27 Weeks of Gestation (n = 13)			GA = 28–31 Weeks of Gestation (n = 26)			GA = 32–36 Weeks of Gestation (n = 86)		
	FIRS (n = 13)	Non-FIRS (n = 0)	*p*-Value	FIRS (n = 24)	Non-FIRS (n = 2)	*p*-Value	FIRS (n = 38)	Non-FIRS (n = 48)	*p*-Value
Birth weight grams	910.38 (257.92)	-	-	1353.33 (374.01)	1575.00 (459.61)	0.44	2299.2 (553.27)	2711.46 (701.38)	**0.006**
Length at birth cm	33.23 (3.46)	-	-	37.18 (3.38)	39.50 (3.53)	0.39	44.56 (3.32)	46.65 (3.27)	**0.002**
Head circumference cm	23.19 (2.43)	-	-	26.31 (2.29)	27.00 (1.41)	0.55	31.09 (2.39)	32.40 (2.08)	**0.018**
APGAR 1 min	3.00 (1.91)	-	-	5.17 (2.31)	6.50 (0.70)	0.55	7.47 (1.03)	8.31 (1.18)	**0.001**
APGAR 5 min	5.54 (1.85)	-	-	6.79 (2.08)	7.50 (0.70)	0.88	8.42 (0.91)	8.96 (0.74)	**0.003**
Gender Male Female	8 (61.53%)	-	-	15 (57.69%)	1 (3.84%)	0.72	18 (20.93%)	31 (36.04%)	0.10
	5 (38.47%)	-		9 (34.63%)	1 (3.84%)		20 (23.25%)	17 (19.78%)	
Umbilical cord IL-6 (pg/mL)	401.61 (548.16)	-	-	332.92 (527.60)	2.23 (1.13)	**0.006**	159.14 (186.35)	4.27 (2.28)	**0.001**
IL-6 at 24 h (pg/mL)	39.77 (44.58)	-	-	47.81 (65.87)	32.59 (3.79)	0.30	51.78 (78.07)	21.82 (31.23)	**0.001**
CRP at 24 h (mg/L)	4.41 (7.18)	-	-	7.69 (25.41)	3.32 (0.96)	0.22	4.99 (8.73)	2.16 (2.72)	**0.03**
PCT at 24 h (ng/mL)	21.49 (33.23)	-	-	22.02 (31.53)	8.08 (10.88)	0.61	19.08 (23.30)	9.28 (11.27)	0.11
EOS	13 (100%)	-	-	21 (95.45%)	1 (4.54%)	0.15	28 (93.33%)	2 (6.67%)	**0.001**
LOS	10 (100%)	-	-	3 (100%)	0 (0%)	0.59	0 (0%)	0 (0%)	-
Respiratory distress	13 (100%)	-	-	24 (92.30%)	2 (7.70%)	-	18 (66.66%)	9 (33.34%)	**0.005**
BPD	10 (100%)	-	-	8 (88.88%)	1 (11.12%)	0.63	0 (0%)	0 (0%)	-
NEC	3 (100%)	-	-	2 (100%)	0 (0%)	0.67	1 (100%)	0 (0%)	0.25
PDA	10 (100%)	-	-	13 (92.85%)	1 (7.15%)	0.91	10 (66.66%)	5 (33.34%)	0.054
ROP	7 (100%)	-	-	6 (100%)	0 (0%)	0.42	2 (100%)	0 (0%)	0.10
IVH/PVL	10 (100%)	-	-	16 (94.11%)	1 (5.89%)	0.63	7 (58.33%)	5 (41.67%)	0.28
Apnea	12 (100%)	-	-	23 (95.83%)	1 (4.17%)	**0.01**	10 (58.82%)	7 (41.18%)	0.17
Feeding intolerance	12 (100%)	-	-	21 (91.30%)	2 (8.70%)	0.59	19 (59.37%)	13 (40.63%)	**0.002**
Seizures	5 (100%)	-	-	4 (100%)	0 (0%)	0.53	3 (100%)	0 (0%)	**0.04**
Bleeding	4 (100%)	-	-	8 (100%)	0 (0%)	0.32	3 (100%)	0 (0%)	**0.04**
Death	3 (100%)	-	-	1 (100%)	0 (0%)	0.76	0 (0%)	0 (0%)	-
Funisitis	12 (100%)	-	-	10 (90.90%)	1 (9.10%)	0.81	11 (78.57%)	3 (21.43%)	**0.005**
Chorioamnionitis	12 (100%)	-	-	13 (92.85%)	1 (7.15%)	0.91	12 (75%)	4 (25%)	**0.006**

## Data Availability

All supporting data are included in this article.
